# Intercostal Artery Pseudoaneurysm Formation after Irinotecan Transarterial Chemoembolization of a Spinal Metastasis from Colorectal Cancer

**DOI:** 10.1155/2012/146540

**Published:** 2012-12-17

**Authors:** Natanel Jourabchi, Steven Sauk, Cheryl Hoffman, Edward Wolfgang Lee, Stephen Thomas Kee

**Affiliations:** ^1^Department of Radiology, David Geffen School of Medicine, UCLA Health System, Los Angeles, CA 90095, USA; ^2^Mallinckrodt Institute of Radiology, Washington University, Saint Louis, MO 63110, USA

## Abstract

Over the past decade, irinotecan has become one of the first-line chemotherapeutic agents used in the treatment of metastatic colorectal cancer. Recently, irinotecan has been administered transarterially in order to perform chemoembolization in the liver. In the limited number of reports available to date using this approach, serious adverse effects have not yet been reported. In this paper, we describe the formation of an intercostal artery pseudoaneurysm after transarterial chemoembolization with irinotecan-eluting beads in a patient with spinal metastasis from colorectal cancer.

## 1. Introduction

Approximately 20% of patients with colorectal cancer (CRC) present with metastatic disease at the time of diagnosis [1]. Currently, surgical resection is the standard of care for patients with isolated metastases from CRC, improving 5-year survival rates up to 32.5% from 10.5% [1]. However, surgical resection can only be performed in 10% of patients [1]; therefore converting initially unresectable metastases into resectable metastases is a crucial step in the management of patients with metastatic CRC. For the past ten years, 5-fluorouracil (5-FU) and oxaliplatin have been used as first-line therapies [2] in the downstaging of metastatic CRC, although recently newer drugs such as irinotecan have been shown to be effective [2].

Irinotecan (Camptosar) is a chemotherapeutic drug that has become more popular over the past decade due to its significant antitumoral activity against metastatic colorectal cancer, although major side effects have limited its systemic use [3]. To minimize the side effects, transarterial chemoembolization with drug-eluting beads has been used instead. To our knowledge, there has been no clinical report of irinotecan chemoembolization for the treatment of metastatic CRC to the spine in patients who are not amenable to surgical resection or radiofrequency ablation. In addition, there has been no clinical report of major side effects in using irinotecan chemoembolization in the treatment of CRC metastases to the spine. In this paper, we present a case of an intercostal artery pseudoaneurysm that developed after chemoembolization with irinotecan-eluting beads for a spinal metastasis of colorectal cancer.

## 2. Case Presentation

**Figure 1:**
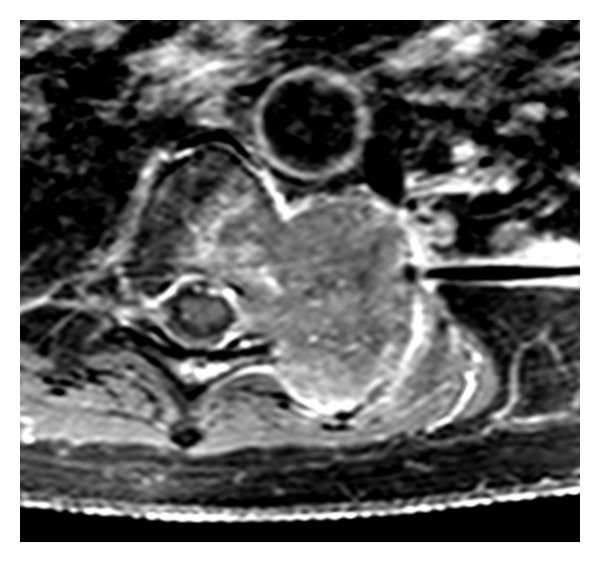
T1 post-Gadolinium fat-saturation MRI T-spine. Note the metastatic tumor in the left paravertebral/chest wall area extending into the epidural space with destruction of the left transverse processes, pedicles, and vertebral bodies. Mass measures 4.0 cm in width × 5.8 cm cephalocaudally × 4.3 cm anterior-posterior direction.

Institutional review board (IRB) approval was obtained for this case presentation. Two months prior to presentation at our institution, a 44-year-old Iranian male was diagnosed with adenocarcinoma of the colon. Initially, the patient received a low anterior resection and lymph node dissection which demonstrated extension into the peritoneal surface with lymph nodes positive for disease (stage T3N1). After being seen in January 2001, the patient underwent chemotherapy with 5-flurouracil, leucovorin, and irinotecan for six months. But in September 2002, his CT scan demonstrated a left lung nodule which was resected in October 2002 and diagnosed as metastatic adenocarcinoma of the colon. Postoperatively, the patient received a second regimen of chemotherapy with 5-flurouracil, leucovorin, and oxaliplatin for four months. Colonoscopy and serial CT scans in 2003 did not demonstrate any recurrent disease. But in February 2004, recurrent bilateral pulmonary nodules were detected and surgically resected which demonstrated again metastatic adenocarcinoma of the colon. A followup CT in November 2004 demonstrated a left pleural mass and bilateral lung nodules, and the patient was started on a third regimen of chemotherapy including 5-fluorouracil, leucovorin, irinotecan, and abciximab. In March 2006, these lesions demonstrated progression which led to a left chest wall resection with a portion of the lung. The lesions on the right were planned for radiofrequency ablation therapy, but in January 2007, the focus shifted to treating a large paravertebral mass involving the left T7, T8, and T9 vertebral bodies revealed on CT and MRI, which was invading the neural foramina with epidural extension (Figure 1). As the tumor extended into the epidural space, surgical removal and radiofrequency ablation (RFA) of the mass were not considered options and the patient's spinal metastasis was treated with chemoembolization and radiation therapy [4].

On the day of the chemoembolization, after written informed consent was obtained, the patient was prepped in a normal sterile fashion. A 5 Fr sheath was placed in the right femoral artery. An angled Glidewire (Meditech, Watertown, MA), Mikaelsson catheter (Angiodynamics, Queensbury, NY), Progreat catheter (Terumo, Leuven, Belgium), and Transend wire (Boston Scientific Target, Fremont, CA) were used to sequentially select multiple intercostal arteries bilaterally. 

**Figure 2:**
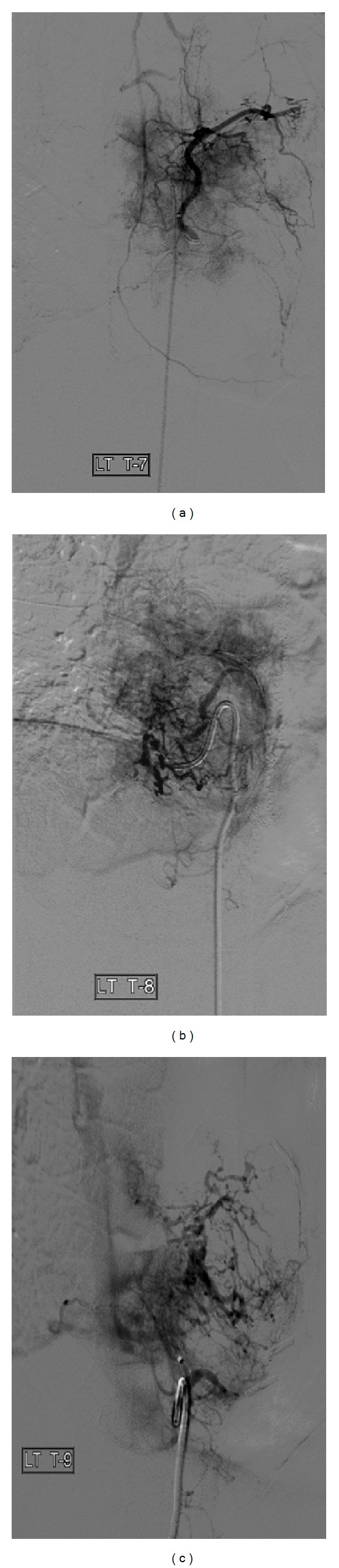
Before TACE with irinotecan-eluting beads. Selections of the (a) left T7 intercostal artery, (b) left T8 intercostal artery, and (c) left T9 intercostal artery. Note the small branches from these intercostal arteries supplying the spinal metastasis from colorectal cancer.

**Figure 3:**
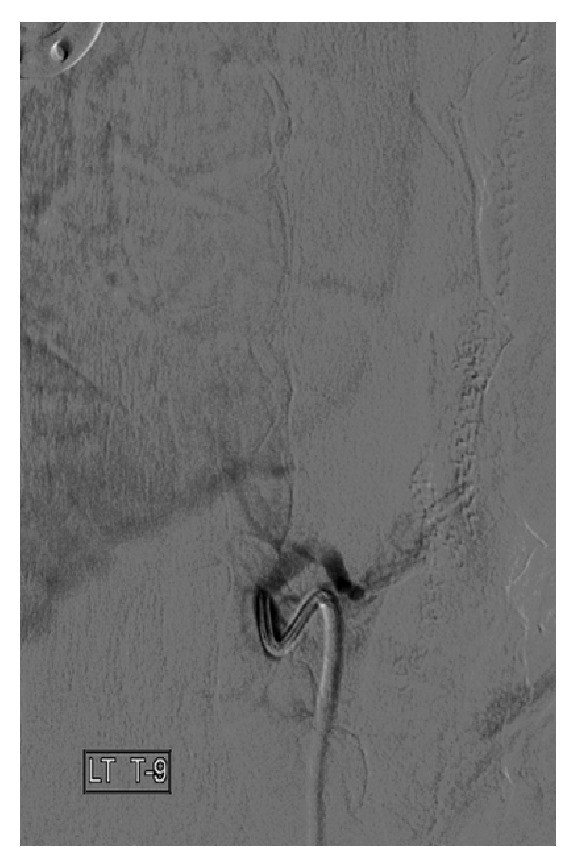
Left T9 intercostal artery immediately after TACE with irinotecan-eluting beads. Note that there is a small branch off of the T9 intercostal artery which still seems to supply the tumor, indicating the need to repeat the procedure in two weeks to completely chemoembolize the branch with irinotecan-eluting beads.

After identifying the feeding vessels of the left paravertebral tumor, which were the left T7, T8, and T9 intercostal arteries, a total of 60 mg of irinotecan suspended in 300–500 *μ*m LC Beads (Biocompatibles, Farnham, Surrey, UK) was injected in divided doses between the left T7, T8, and T9 vertebral arteries. Due to vasospasm, only partial embolization of the T9 intercostal artery was accomplished (Figures 2 and 3). Therefore, the patient was scheduled to return two weeks later to receive additional chemoembolization of this particular vessel.

**Figure 4:**
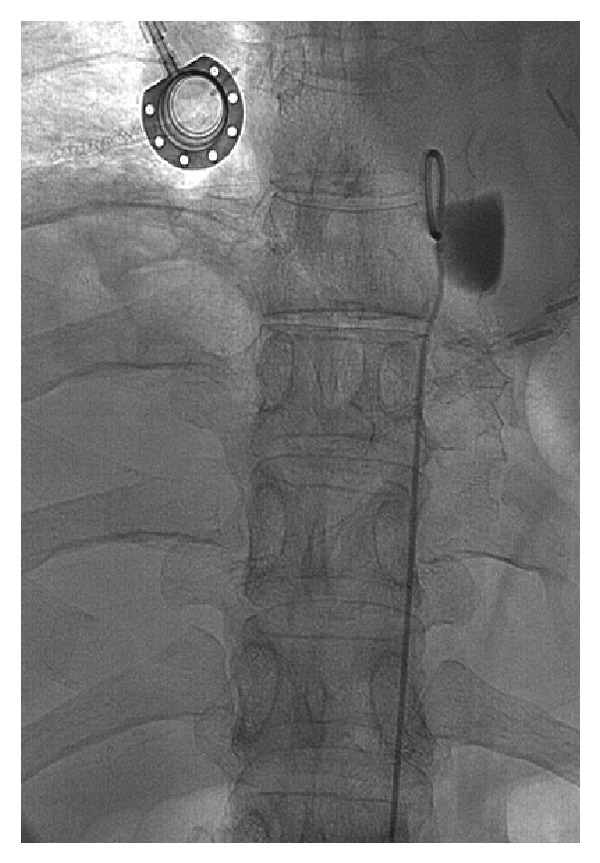
Two weeks after chemoembolization. Selection of the T8 intercostal artery. Note that a laterally projecting outpouching of stagnant contrast was noted, consistent with pseudoaneurysm. The opacifying portion of the pseudoaneurysm measured about 2-3 cm in size with a 5 mm neck.

Two weeks later with the intention of embolizing the T9 intercostal artery, reintervention was performed. The patient was prepped in a normal sterile fashion, and a 5 Fr sheath was inserted into the right common femoral artery. A 5 Fr Mikaelsson spinal catheter was then advanced into the left T8 through T10 intercostal arteries and the right T8 intercostal arteries. The left T9 intercostal artery angiogram demonstrated that the previously noted small feeding arteries to the known tumor were no longer visible likely because of spontaneous thrombosis. However, in the proximal left T8 intercostal artery, a laterally projecting outpouching of stagnant contrast was noted, consistent with a pseudoaneurysm (Figure 4). Although a contained aortic dissection was considered, in real time, the focal outpouching of contrast arose from the left T8 intercostal artery and not from the aorta. The opacifying portion of the pseudoaneurysm measured about 2-3 cm in size with a 5 mm neck.

**Figure 5:**
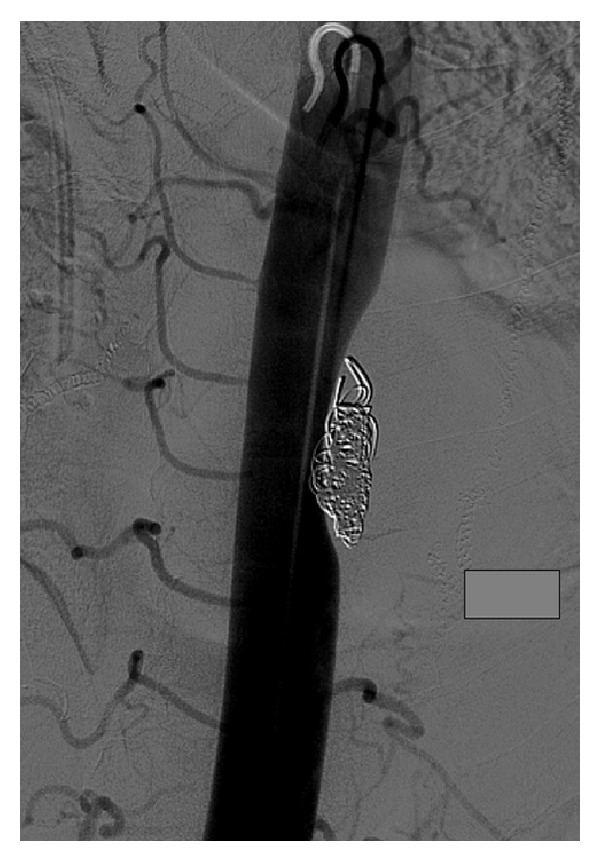
Aortogram, after coil embolization of the T8 intercostal artery. Note that the postcoil embolization aortoagram demonstrates no contrast opacification of the pseudoaneurysm lumen, a coil mass measuring approximately 1.5 × 3.5 cm, and a gentle regional concave deformity of the aorta, implying that the true lumen of the pseudoaneurysm was larger than what opacified originally.

**Figure 6:**
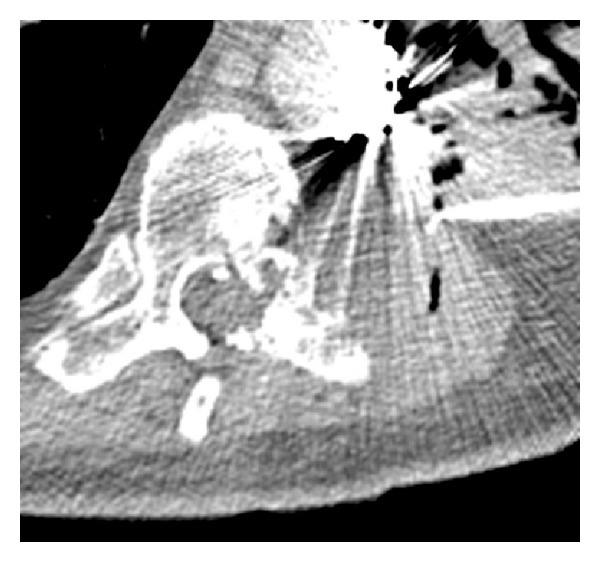
CT T-spine without contrast five months after irinotecan TACE and after pseudoaneurysm coil embolization. Note the metastatic lesion in the left paravertebral/chest wall area which has decreased in size, now measuring 5.5 cm × 5.2 cm.

The pseudoaneurysm was embolized with a microcatheter system. Embolization was achieved with 16 GDC 360 coils and 5 MTI Helix coils, and the postembolization aortogram demonstrated (1) no contrast opacification of the pseudoaneurysm lumen, (2) a coil mass measuring approximately 1.5 × 3.5 cm, and (3) a gentle regional concave deformity of the aorta, implying that the nonthrombosed pseudoaneurysm sac of the pseudoaneurysm was larger than what opacified originally (Figure 5). The patient had an uneventful hospital course following the procedure and was discharged the following day. Although the patient had noted having new low grade left-sided chest discomfort for two weeks between the first two chemoembolization procedures, upon followup one month later, the patient reported that his chest discomfort had resolved. In addition, his CEA decreased from 11 ng/mL before procedure to 5.5 ng/mL after procedure. Postembolization imaging demonstrated a decrease in size of the spinal metastasis from 6.6 cm × 6.2 cm on preprocedure CT to 5.5 cm × 5.2 cm on postprocedure CT (Figure 6).

## 3. Discussion

Irinotecan (Camptosar) is a chemotherapeutic drug that has become more popular over the past decade due to its significant anti-tumoral activity against metastatic colorectal cancer [3]. It inhibits cancer growth primarily by inhibiting topoisomerase I in actively dividing tumor cells [3]. When used separately, the efficacy of irinotecan was comparable to 5-FU; used together, synergistic improvement in efficacy was shown [5]. Despite the improvements in the overall survival of patients treated with irinotecan, major side effects such as grade 3-4 diarrhea and neutropenia [6, 7] limit the systemic use of irinotecan [3]. 

To minimize the side effects, transarterial chemoembolization with drug-eluting beads has been used. With chemoembolization, there is a significant reduction of these systemic side effects with only mild abdominal and shoulder pain, vomiting and mild asthenia, and some alopecia [6]. No serious side effects such as diarrhea and neutropenia were reported, supporting the premise that this approach is better tolerated when irinotecan is given transarterially with drug-eluting beads than when administered systemically. A study of chemoembolization with irinotecan-eluting beads in treatment of metastatic CRC demonstrated a 50–90% reduction of tumor marker carcinoembryonic antigen (CEA), significant reduction of contrast enhancement on postprocedure CT imaging, and downstaging of CRC metastases [6]. However, the long-term effects of irinotecan chemoembolization on these patients are not yet known.

Several papers have demonstrated the palliative effects of chemoembolization in the treatment of spinal metastases from various primary cancers for patients who failed systemic chemotherapy, are not amenable to surgical resection or radiofrequency ablation of their spinal tumors, and have a dominant soft tissue component that precludes treatment with acrylic cement [8, 9]. The most recent study by Chiras et al. demonstrated an 83% success rate in pain relief after selective intra-arterial chemotherapy with carboplatin and chemoembolization with pirabucin for metastatic kidney, breast, lung, and gastrointestinal cancers and sarcomas to the spine [8]. No major side effects were reported. 

The use of irinotecan in combination with 5-FU/folinic acid as a first- and second-line systemic treatment of advanced colorectal cancer is on the rise [6, 7, 10]. Although systemic dosing is limited by intolerable toxicities, mainly of the gastrointestinal and hematologic systems, chemoembolization directs systemic doses on the targeted site. Therefore, delivering chemotherapeutic drugs using chemoembolization provides the benefit of concentrating therapeutic doses directly on the lesions while avoiding intolerable systemic side effects. 

As mentioned previously, the long-term outcome of irinotecan chemoembolization with drug-eluting beads is still pending. The preliminary trial of chemoembolization with irinotecan-eluting beads on colorectal cancer metastases to the liver by Aliberti et al. demonstrated a 50–90% decrease in CEA levels and a decrease in lesional contrast enhancement [6]. Five months after irinotecan chemoembolization, our patient's CEA level decreased by 50%, and a decrease in the size of the spinal metastasis was seen. The reported side effects of the procedure from the Aliberti et al. data when treating liver metastases, including abdominal pain, shoulder pain, vomiting, mild asthenia, and alopecia, were not experienced by our patient undergoing spinal metastasis therapy at three months after procedure [6]. 

However, our patient experienced a side effect of a pseudoaneurysm formation after chemoembolization with irinotecan. This is a rare and interesting report of a pseudoaneurysm after chemoembolization with irinotecan. Our patient received approximately 20 mg of irinotecan-loaded beads in each of the three chemoembolized intercostal arteries (T7–T9). Two weeks after the procedure, a pseudoaneurysm was discovered in the proximal segment of the T8 intercostal artery. 

The overall incidence of intercostal artery pseudoaneurysms is rare, and only seven cases have been reported in the literature [11–17]. Five of these cases were post-surgical and two were traumatic. Although uncommon, the occurrence of a pseudoaneurysm after chemoembolization represents a risk that can threaten the patient's outcome, especially if the pseudoaneurysm ruptures.

Laboratory evidence of vascular damage from chemotherapeutic drugs for metastatic CRC has been reported only for doxorubicin. At high doses, doxorubicin compromises the vascular wall by causing excoriation and apoptosis of endothelial and smooth muscle cells [18]. Although irinotecan acts differently from doxorubicin, by inhibiting topoisomerase I, there may be similarities in its ability to damage arteries. Gene chip analyses from cancer cells exposed to irinotecan have demonstrated upregulation of proapoptotic genes and downregulation of antiapoptotic genes [19]. This may suggest the possibility that like doxorubicin, irinotecan may compromise the vascular endothelium. In a study of irinotecan-eluting stents in rabbit aortas, high-dose irinotecan also resulted in decreased vessel wall thickness, increased necrosis, and inflammation [20]. Possibly, through a mechanism of proapoptotic gene activation and/or necrosis, irinotecan weakens the vascular wall, thereby increasing the risk of developing an aneurysm. Further studies to investigate irinotecan effect on the vascular wall would therefore be helpful.

The standard treatment for patients with spinal metastases from colorectal cancer is surgical resection and/or RFA. In this case, the tumor had invaded the epidural space, limiting these options. The standard traditional option would have been radiation therapy with bland embolization. The addition of effective chemotherapeutic drugs with embolic material has gained favor for tumors not amenable to surgery or RFA. To date, there is only one study [9] in its preliminary stage demonstrating the long-term effects of chemoembolization with irinotecan-eluting beads and no clinical report of vascular consequences of irinotecan chemoembolizations. The development of a pseudoaneurysm in the T8 intercostal artery secondary to traumatic vessel injury occurring during catheterization cannot be completely excluded. The cannulation, however, appeared atraumatic and was completed by the attending interventional radiologist.

In summary, this case demonstrates the formation of a pseudoaneurysm after transarterial chemoembolization with irinotecan-eluting beads. Further studies are warranted to evaluate the effects of irinotecan on the vascular wall and the possibility that this could affect the long-term clinical outcomes of irinotecan chemoembolization.
